# Exosomes Derived From Pericytes Improve Microcirculation and Protect Blood–Spinal Cord Barrier After Spinal Cord Injury in Mice

**DOI:** 10.3389/fnins.2019.00319

**Published:** 2019-04-16

**Authors:** Xiaochen Yuan, Qingbin Wu, Peng Wang, Yingli Jing, Haijiang Yao, Yinshan Tang, Zhigang Li, Honggang Zhang, Ruijuan Xiu

**Affiliations:** ^1^Key Laboratory of Microcirculation, Ministry of Health, Institute of Microcirculation, Chinese Academy of Medical Sciences & Peking Union Medical College, Beijing, China; ^2^Orthopedics Department, Hebei Provincial Hospital of Traditional Chinese Medicine, Shijiazhuang, China; ^3^China Rehabilitation Science Institute, China Rehabilitation Research Center, Center of Neural Injury and Repair, Beijing Institute for Brain Disorders, Beijing Key Laboratory of Neural Injury and Rehabilitation, Beijing, China; ^4^Treatment Center of TCM, Beijing Bo’ai Hospital, China Rehabilitation Research Center, School of Rehabilitation, Capital Medical University, Beijing, China; ^5^Department of Rehabilitation in Traditional Chinese Medicine, The Second Affiliated Hospital, Zhejiang University School of Medicine, Hangzhou, China; ^6^School of Acupuncture, Moxibustion and Tuina, Beijing University of Chinese Medicine, Beijing, China

**Keywords:** pericytes, exosomes, spinal cord injury, microcirculation, microvascular

## Abstract

Spinal cord injury (SCI) often leads to severe and permanent paralysis and places a heavy burden on individuals, families, and society. Until now, the therapy of SCI is still a big challenge for the researchers. Transplantation of mesenchymal stem cells (MSCs) is a hot spot for the treatment of SCI, but many problems and risks have not been resolved. Some studies have reported that the therapeutic effect of MSCs on SCI is related to the paracrine secretion of cells. The exosomes secreted by MSCs have therapeutic potential for many diseases. There are abundant pericytes which possess the characteristics of stem cells in the neurovascular unit. Due to the close relationship between pericytes and endothelial cells, the exosomes of pericytes can be taken up by endothelial cells more easily. There are fewer studies about the therapeutic potential of the exosomes derived from pericytes on SCI now. In this study, exosomes of pericytes were transplanted into the mice with SCI to study the restoration of motor function and explore the underlying mechanism. We found that the exosomes derived from pericytes could reduce pathological changes, improve the motor function, the blood flow and oxygen deficiency after SCI. In addition, the exosomes could improve the endothelial ability to regulate blood flow, protect the blood-spinal cord barrier, reduce edema, decrease the expression of HIF-1α, Bax, Aquaporin-4, and MMP2, increase the expression of Claudin-5, bcl-2 and inhibit apoptosis. The experiments *in vitro* proved that exosomes derived from pericytes could protect the barrier of spinal cord microvascular endothelial cells under hypoxia condition, which was related to PTEN/AKT pathway. In summary, our study showed that exosomes of pericytes had therapeutic prospects for SCI.

## Introduction

Spinal cord injury (SCI) refers to the most common and destructive injuries in spinal surgery which affects about 180,000 patients all over the world every year (Chopp et al., 2000). SCI often leads to severe and permanent paralysis, accompanied by a significant decline in the quality of life, placing a heavy burden on individuals and their families. SCI is characterized by rapid triggering of secondary damage after primary mechanical damage. Ischemia, hypoxia, inflammatory reactions, apoptosis of neurons, and oligodendrocytes appear in the damage area. Over time, astrocyte scarring and spinal cord voids form which inhibit nerve axon regeneration and result in the motor and sensory dysfunction below the damage plane (Schwab and Bartholdi, 1996). At present, SCI treatment is still the biggest challenge for researchers (Carlson and Gorden, 2002).

With the development of stem cell technology, stem cell transplantation has become a hot topic for the treatment of SCI. MSCs possess the multipotency and can be induced into many kinds of cells, such as osteoblasts (bone cells), chondrocytes (cartilage cells), myocytes (muscle cells), and adipocytes (Ho et al., 2008). Some studies have proved that transplantation of MSCs after SCI may be a promising strategy to improve the functions of motor, sensory and/or autonomic nerves (Chopp et al., 2000; Feron et al., 2005; Cizkova et al., 2011; Mothe and Tator, 2012; Nakajima et al., 2012; Neirinckx et al., 2015). However, several studies have shown that the stem cells have lower survival rates in tissues and there are some risks such as cell de-differentiation, immune rejection and malignant tumor formation after transplantation (Balsam et al., 2004; Rubio et al., 2005; Jeong et al., 2011; Rodriguez et al., 2012). Recently, the increasing evidence has proved that the curative effects of MSCs are mostly due to paracrine mechanism, with exosomes taking a great part in this process (Katsuda et al., 2013; Ratajczak et al., 2014). Many kinds of cells can produce exosomes which are small membrane vesicles of internal origin about 30–120 nm in diameter (Stoorvogel et al., 2002). and contain functional mRNA and microRNA, as well as proteins (Record et al., 2011). Exosomes are important for cell communication and suitable to deliver small RNA and proteins (Mathivanan et al., 2010; Zomer et al., 2010). The specific surface ligands of the exosomes allow them to bind the target cells, transmit biological information and related genetic proteins to the target cells, and eventually regulate specific biological functions of cells (Breakefield et al., 2011; Chaput and Théry, 2011). Some studies have shown that exosomes and microvesicles of MSCs can be used for the treatment of liver, cardiovascular, and kidney injury (Gatti et al., 2011; Lai et al., 2011; Li et al., 2012). Studies have shown that the exosomes of MSCs have therapeutic effect on SCI in rats (Huang et al., 2017; Liu et al., 2018).

Pericytes (also known as Rouget cells) are contractile. They encircle the endothelial cells that line the capillaries and venules throughout the body (Simonavicius et al., 2012). They are also important ingredients of the neurovascular unit containing neurons, endothelial cells and astrocytes (Dore-Duffy and Cleary, 2011). Pericytes and endothelial cells establish a close link by multiple intercellular connection and sharing the same basement membrane. Pericytes participate in the control of pressure of microcirculation, integrity and permeability of microvascular, and take part in angiogenesis and differentiation regulation of endothelial cells by direct physical contact and paracrine signaling (Bergers and Song, 2005; Orekhov et al., 2014). Pericytes are heterogeneous, which express MSCs specific markers including CD44, CD73, CD90, CD105, and CD146, platelet-derived growth factor receptor-β (PDGFR-β), stromal precursor antigen-1 (STRO-1), neural glial antigen (NG2), and alpha smooth muscle actin (α-SMA). Due to the special relationship between pericytes and endothelial cells, endothelial cells are able to take up exosomes of pericytes which participate in the mediation of endothelial function. There are fewer studies about therapeutic potential of the exosomes derived from pericytes on SCI. In this study, pericytes exosomes were transplanted into the mice with SCI in order to study their functional recovery and explore the underlying mechanism.

## Materials and Methods

### Experimental Animals

Male ICR mice (License No. SCXK2014-0004) of 8-week-old were bought from the Institute of Laboratory Animal Science, Chinese Academy of Medical Sciences (CAMS) & Peking Union Medical College (PUMC). The mice were bred at 26°C, 38.5% humidity, with a 12-hr light, 12-hr dark cycle (7:30 a.m–7:30 p.m light).

### Animal Welfare

The protocols of animal experiments were approved by the Experimental Animal Care and Ethics Committee of the Institute of Microcirculation, CAMS & PUMC.

### Experimental Design

#### In vivo

A total of 90 mice. 30 mice were randomly selected as sham group (Sham group) without SCI. 60 mice were randomly divided into two groups after successful building SCI model: a phosphate-buffered saline (PBS) treatment group (SCI group) and a pericytes exosomes treatment group (Exosomes group). Mice were subjected to SCI and then followed by tail vein injection of exosomes (20 μg of exosomes in 0.3 mL PBS or 0.3 mL PBS) starting an hour after contusion SCI was induced. After 48 h of surgery, 5 mice from each group were detected by Laser Doppler and then the spinal cord was taken out for WB assay. 20 mice in each of the three groups were arranged to immune- histochemistry (*n* = 5), assessment of microvascular permeability of spinal cord (*n* = 10), evaluation of spinal cord edema (*n* = 5). The remaining 5 mice in each group were performed a behavioral study on the 14th day after the injury.

#### In vitro

Isolation and culture of primary ICR mouse spinal cord microvascular endothelial cells (SCMECs). Cells were cultured under three conditions: Normal conditions, hypoxic conditions, hypoxia + exosomes (20 μg/ml final concentration). The permeability of monolayer endothelial cells was detected by Transwell system (Corning, Union City, CA, United States). The cultured cells were collected for further experiments.

The experiment was conducted under double-blind condition to avoid potential biases of performance and detection.

### Isolation of Micro-Vessels, Culture and Identification of Pericytes

Mice were anesthetized using pentobarbital sodium (100 mg/kg body weight). The brains of the ICR mice were removed and immersed in ice-cold isolation buffer, micro-vessels were isolated as previously reported (Yuan et al., 2018). After removal of the vessels. the gray matter of the brain was isolated and minced in ice-cold Dulbecco’s modified Eagle’s medium (DMEM), under a dissecting microscope. Then the tissues were digested in DMEM containing collagenase type II (1 mg/ml), DNase I (15 μg/ml) and gentamicin (50 μg/ml) at 37°C for 1.5 h and centrifugated in 20% bovine serum albumin (BSA)/DMEM (1000 ×*g*, 20 min). Then they were further digested with collagenase/dispase (1 mg/ml; Roche, Switzerland) and DNaseI (6.7 μg/ml) in DMEM at 37°C for 1 h. The micro-vessel clusters were separated using 33% continuous Percoll (GE Healthcare, United Kingdom) gradient (1000 ×*g*, 10 min), and washed twice in DMEM. The microvessel fragments were cultured in DMEM supplemented with 10% fetal bovine serum (FBS), 100 U/ml penicillin, and 100 μg/ml streptomycin. After microvessel adherence (48 h), fresh medium (DMEM supplemented with 10% FBS, 100 U/ml penicillin, and 100 μg/ml streptomycin) was replaced, floating dead cells and other impurities were removed, and then medium was replaced every 2 days. The pericytes were used for subsequent experiments when they reached 80–90% confluence. Pericytes were identified by surface markers desmin (1:250; ab15200; Abcam) and α-SMA (1:250; ab21027; Abcam). Immunocytochemistry with Von Willebrand Factor (vWF, 1:200; ab11713; Abcam) was carried out as previously described (Wu et al., 2015) to determine whether there was endothelial cell contamination.

### Isolation of Micro-Vessels and Culture of SCMECs

Mice were anesthetized using pentobarbital sodium (100 mg/kg body weight). The spinal cord of the mice was removed from canalis vertebralis and placed in ice-cold isolation buffer. The micro-vessel fragments were isolated as above described and cultured in DMEM supplemented with 10% FBS, 100 U/ml penicillin, 100 μg/ml streptomycin and 4 μg/mL Puromycin. After 48 h, culture medium without purinomycin was replaced. Then the culture medium was changed every 2 days. The endothelial cells were used for subsequent experiments when they reached 80–90% confluence. The cells that did not express permeability glycoprotein (P-gp) should be killed by puromycin after treatment for 48 h while endothelial cells expressed P-gp, so endothelial cells with high purity could be obtained.

### Pericytes Exosomes Generation and Collection

When pericytes reached 60–80% confluence, they were cultured in an exosomes depleted FBS-contained (EXO-FBS-250 A-1; System Biosciences, Mountain View, CA, United States) medium, for an additional 48 h. Then the medium weas gathered and exosomes were isolated by multi-step centrifugation, as previously reported (Xin et al., 2012; Villarroya-Beltri et al., 2013). Briefly, supernatants collected from cultured pericytes were centrifuged at 2000 *g* for 30 min to get rid of the large debris and dead cells, at 10,000 *g* for 30 min to remove the small-cell debris and then at 100,000 *g* for 70 min. At last, contaminating proteins was removed by centrifugation at 100,000 *g* for 70 min. Exosomes were saved at -80°C or utilized for another series of experiments. Exosomes protein content was examined using a bicinchoninic acid assay (BCA). Western blotting was used to examine the specific exosome surface markers which was encapsulated into exosomes including CD9 (1:1000; ab92726; Abcam) and CD81 (1:1000; ab109201; Abcam).

### Transmission Electron Microscopy of Pericytes Exosomes

The morphology of pericytes exosomes was observed by transmission electron microscopy (TEM). Exosomes pellets were fixed in 2% paraformaldehyde (PFA) -cacodylate buffer and then they were loaded to copper grids covered with formvar for 20 min. Then exosomes were fixed in 1% (w/v) glutaraldehyde for 5 min. Grids were washed and contrasted in 4% uranyl acetate for 5 min, dried, and observed by TEM (FEI TECNAI G2,120 KV).

### Size Distribution Analysis of Pericytes Exosomes

The suspensions with vesicles were analyzed by Nano-Sight LM10 instrument (Malvern, Worcestershire, United Kingdom). A monochromatic laser beam lightened the diluted samples at 405 nm to record a 60 s video taken with a mean frame rate of 25 frames/s. EVs samples were analyzed by the NTA software (version 3.0, Nano-Sight) to distinguish firstly and then followed up each particle on a frame-by-frame basis optimally, and Brownian movement of it was tracked and measured from frame to frame. The size of particle was determined with the two-dimensional Stokes-Einstein equation on basis of the velocity of particle movement. The mean, mode, and median EVs size from each video was used to calculate samples concentration expressed in nanoparticles/mL.

### Building of SCI Models

Animals were anesthetized by inhalation of 1.5% isoflurane and performed laminectomy at thoracic vertebra level 10 (T10) on a calorstat heating pad at the prone position. Briefly, a laminectomy was carried out at the T10 level, and we clamped the spinous processes of T8 and T11 in order to stabilize the spine. 50-kd spinal contusion injury was made in mice with the Infinite Horizons Impactor (Precision Systems and Instrumentation, Lexington, KY, United States) (Allen, 1911; Yuan et al., 2017). Mice in the sham group were only performed laminectomy without impact. Then the animals were put in a warming chamber at about 38.5°C until they woke up completely. The bladders of mice were manually emptied twice a day until the mice were able to recover autonomic bladder function during this period. After surgery, buprenorphine (0.05 mg/kg, ip) was administered at once and then every 6 h for 1 day to reduce pain.

### Behavioral Study

Functional recovery after SCI was determined by the Basso Mouse Scale (BMS) scores (Basso et al., 2006). Mice were tested on postoperative days 1, 3, 5, 7, 10, and 14 for the duration of the experiments. Then the scores were recorded in an open-field environment by trained investigators under double-blind conditions.

### Tissue Processing

After 48 h of injury, mice were anesthetized and perfused transcardially with 0.9% saline (containing 50 U/mL heparin), followed by 4% PFA in phosphate buffer. 10 mm spinal cord segments were taken with the injured site as the center and placed in the same fixative for 48 h at room temperature. The specimens were embedded into paraffin for production of 5-μm-thick transverse sections at the sites 500 μm rostral to the lesion epicenter. There were three sections used for each immunostaining per mice.

### Immunohistochemistry

The sections were incubated in 0.3% hydrogen peroxide for 30 min and in 0.1% Triton X-100 for 20 min. Then they were incubated with anti-Aquaporin-4 (AQP4) antibody (1:100; ab9512; Abcam), with anti-MMP-2 antibody (1:200; ab86607; Abcam), or with anti-claudin-5 antibody (1:200; ab15106; Abcam) overnight at 4°C and washed with PBS, incubated with secondary antibody, at 37°C for 60 min. At last, the slices were washed with PBS and sealed with the coverslip.

### Luxol Fast Blue (LFB) Staining

The slices were placed in a Luxol fast blue solution (Servicebio^®^, China), incubated overnight at 57°C, rinsed with 95% ethanol and distilled water for 3 min, respectively, differentiated in 0.05% lithium carbonate solution for 15 s, and placed in 70% ethanol to continue to differentiate for 30 s until the gray matter was clearly identifiable.

### Nissl Staining

The sections were de-paraffinized in xylene 2 or 3 for 10 min each. Then they were hydrated in 100% alcohol for 2 × 5 min followed by 95% alcohol for 3 min, 70% alcohol for 3 min. Next, the sections were rinsed in tap water and then in distilled water. At last they were stained in 0.1% cresyl violet solution for 3–10 min, rinsed quickly in distilled water, differentiated in 95% ethyl alcohol for 2–30 min and checked microscopically for best result. The sections were dehydrated in 100% alcohol for 2 × 5 min, and cleared in xylene for 2 × 5 min and mounted with permanent mounting medium.

### Terminal Deoxynucleotidyl Transferase-Mediated dUTP-Biotin Nick End Labeling (TUNEL) Staining

As for apoptosis detection on injury site, terminal deoxynucleotidyl transferase-mediated dUTP nick end labeling (TUNEL) staining was applied using the *in situ* Cell Death Detection Kit (Roche, Mannheim, Germany). The process was done according to manufacturer’s instructions. The sections were dewaxed, rehydrated, and washed. Then the sections were pre-treated with proteinase-K for 30 min and incubated with TUNEL reaction mixture for 60 min at 37°C. The converter POD was added and incubated for 30 min at 37°C. The sections were washed with PBS and incubated with diaminobenzidine for 10 min. For quantitative analysis, the sum of the positively stained cells from five random visual fields of the anterior horn of the gray matter were calculated.

### Laser Doppler Imaging (LDI) Measurement of Spinal Cord Blood Perfusion

Microvascular blood flow (MVBF) of spinal cord was examined using the Laser Doppler Line Scanner^®^ (LDLS; Moor Instruments, Axminster, United Kingdom) at a stable temperature (24 ± 1°C) and 60% relative humidity. A line of 785 ± 10 nm laser light was used to scan over spinal cord of anesthetized mice. A scanning mirror along with optics on a 64-element linear array could direct the doppler shifted or non-shifted light from the moving blood cells or fixed tissue, respectively, and then establish a two-dimensional color-coded perfusion image. Data was computerized and recorded as image and numerical data (perfusion units, PU). For LDI analysis, three flux images were obtained through continuous scan. Thereafter, in moor LDI Image Review, version 5.3 (Moor Instruments Ltd.), the images were averaged to minimize any disturbance caused by movements (Rendell et al., 1999).

### Detection of Vasomotion of Spinal Cord and Spectral Analysis of Laser Doppler Flowmetry (LDF) Signal

After survey of blood perfusion, we measured the vasomotion by dual channel Laser Doppler monitor (Moor – VMS – LDF2) instrument (Moor Instrument, Ltd., Axminster, United Kingdom) and a fiberoptic probe (Moor Instruments) with a calculated penetration depth of 2.5 mm. The electrode was placed within 1 mm to the detection site. After each run, the probes were replaced to shun additive effects and partial exhaustion of contractive and relaxative ability. A specific device containing colloidal latex particles was used to calibrate data before each test session. The normalized values were supplied by brownian motion of these particles The LDF signal was documented consecutively by the interfaced computer equipped Moor software for Windows version (Moor VMS – PC 2.0, Moor Instrument) as previously described (Rendell et al., 1999). Briefly, as for the analysis of LDF, a 5 min consecutive data were filtered by a built-in noise filter in the software to remove any noise spikes and frequencies above 10 Hz, which were used for subsequent wavelet analysis. The wavelet analysis was performed by means of the Moor software. A three-dimensional (3D) plot was produced from the wavelet transformations of perfusion signals, which linked representation of vasomotor outputs with the time and frequency domain. Then 3D plot was projected in two dimensions as averaged over time. According to previous reports (Li et al., 2007; Pavlov et al., 2014; Neganova et al., 2017), 0.01–5 Hz was detected and slower contributions (0.01–0.25 Hz) were recognized caused by endothelial factors. The relative value of endothelial factors was recorded as the ratio between the value of endothelial amplitude and the sum amplitudes for total frequency range.

### Assessment of Microvascular Permeability of Spinal Cord

Evan’s Blue (EB) leakage was evaluated using a protocol as described previously (Yuan et al., 2017). EB dye (2% (w/v) in saline; Sigma-Aldrich, St Louis, United States) was infused intraperitoneally. After 3 h, animals were narcotized and infused transcardially with saline. The spinal cord was removed, dried and weighed. The samples were cut into pieces and soaked in a 50% trichloroacetic acid solution for 72 h at room temperature and centrifuged at 10,000 *g* for 10 min. Then we measured the fluorescence of the supernatants using excitation wavelength at 620 nm and emission wavelength at 680 nm. A standard curve with EB dye (0, 50, 100, 200, 400, 800, 1600, 3200, and 6400 ug in trichloacetic acid) was produced and fluorescence intensity was assessed with a spectrophotometer using excitation wavelength at 620 nm and emission wavelength at 680 nm. All data were within the range of detection built by the standard curve. The concentration of dye was qualified as the ratio of absorbance relative to the amount of tissue. Dye of samples was recorded as μg/mg of tissue.

To qualify the EB extravasation, mice were inundated with saline and subsequently with 4% PFA. The spinal cords were sectioned into 20-μm thick with a cryostat. The fluorescence of EB in spinal tissue was observed with a fluorescence microscope and the relative fluorescence intensity was assessed by Image Pro Plus 7.0.

### Evaluation of Spinal Cord Edema

To evaluate edema formation, mice were anesthetized, and T9 (rostral cord, approximately 2 mm), T10 (epicenter, approximately 2 mm), and T11 (caudal cord, approximately 2 mm) segments were immediately removed. The samples (size, 5–15 mg) were quantified at once and put into an oven at 90°C for 72 h to get their dry weights. Edema formation was accounted by water content decided from the difference between the wet and dry weights of the samples, as reported before (Sharma and Olsson, 1990).

### Establishment of Hypoxic Model to Cells

The SCMECs were seeded in 6-well plates or Transwell system (Yuan et al., 2011). When the cells kept a good state and reached 90%, the old mediums were abandoned, and new mediums were added. The cells were divided into three groups: (1) Hypoxic + Exosomes group: the exosomes were added into the medium at concentration of 20 μg/ml, and the cells were cultured in a 37°C tri-gas hypoxic incubator. The culture condition was 5% CO_2_, 94% of N_2_, and 1% of O_2_. (2) Hypoxic group: The cells were cultured in a 37°C tri-gas hypoxic incubator with a culture condition of 5% CO_2_, 94% of N_2_, and 1% of O_2_. (3) Control group: cells were cultured in normal incubator, with a culture condition of 5% CO_2_, 37°C. The culture time was 6 h (Nohda et al., 2007).

### Endothelial Permeability Assay

We assessed the paracellular permeability with a *trans*-well system by measuring the flux of FITC–dextran across the endothelial monolayer. FITC-conjugated dextran (40 kDa, 2 mg/mL; Sigma, St Louis, MO, United States) was put in the upper chamber of the Transwell system. Ten microliters of aliquots was taken away from the lower chamber at 0, 5, 15, 30, 60, or 90 min and changed with fresh medium. At last, the fluorescence that passed through the cell-covered inserts was determined with a fluorescence multiwell plate reader.

### Western Blot Analysis

Total protein was extracted from spinal cord (epicenter ± 5 mm) with a lysis buffer (Beyotime, China). Briefly, at 4°C, tissue homogenates were lysed for 1 h and then centrifuged at 14,000 *g* for 8 min. The protein was qualified by BCA TM assay kit (Pierce, Rockford, IL, United States). Membrane Protein Extraction Kit (Thermo Scientific, Waltham, MA, United States) was used to extract the proteins from SCMECs. 50 μg total protein was separated by 10% SDS–PAGE and transferred to PVDF membranes (Pall Life Sciences, Port Washington, NY, United States). We incubated the membranes with diluted primary antibodies overnight at 4°C. The primary antibodies included Bax (1:1000; ab32503; Abcam), Bcl-2 (1:2000; ab196495; Abcam), HIF-1 (1:1000; ab82832; Abcam), β-actin (1:1000; ab5694; Abcam), PTEN (1:800; ab31392; Abcam), p-akt (1:1000; ab38449; Abcam), ZO-1 (1:1000; ab96587; Abcam). The membranes were washed it with TBST for three times, and then incubated with appropriate horseradish peroxidase-conjugated secondary antibodies for 1 h at 37°C. The bands were detected using enhanced chemiluminescence (ECL).

### Quantitative Measurement of Image and Statistical Analyses

We assessed fluorescence intensity of EB dye, expressions of proteins and the number of apoptosis cells quantitatively with Image Pro Plus 7.0 (Media Cybernetics, Silver Spring, MD, United States). Images of each analytical group were taken from anterior horn of the gray matter or canalis centralis medulla spinalis and acquired using identical exposure settings. The data was analyzed by SPSS version 17.0 statistic software package (Chicago, IL, United States). Significance between SCI and exosomes treated groups was assessed by Student’s *t*-test. Significance in three or more groups was determined by one-way analysis of variance (ANOVA) followed by *post hoc* Tukey’s analysis. All data was presented as mean ± SD, and values of *P* < 0.05 were considered significant.

## Results

### Morphology of Pericytes and Expression of Generic Markers

**FIGURE 1:**
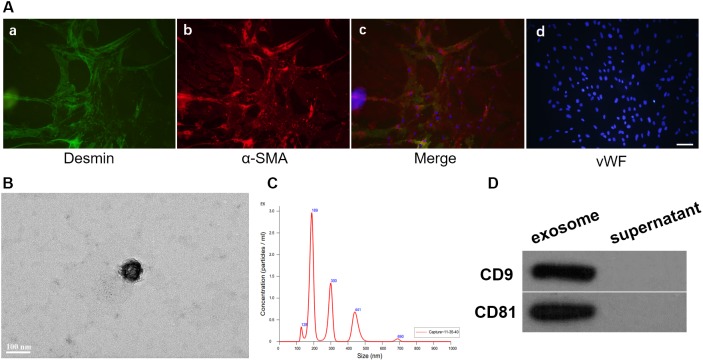
Identification of the pericytes from cerebral micro-vessels and the pericytes exosomes. **(A)** Pericytes were double labeled with desmin and α-SMA, and identified by fluorescence. Pericytes were stained with desmin (a). Pericytes were stained with α-SMA (b). The two types of fluorescence with dapi were merged (c). Pericytes were stained with vWF and dapi (d). Bar = 100 μm. **(B)** Morphology of exosomes was observed by TEM. **(C)** Nanoparticle size distribution was analyzed by Nano-Sight LM10 instrument. **(D)** The surface markers of the exosomes, CD9 and CD81, were analyzed by Western blot.

After 7 days of culture, pericytes climbed out of the brain micro-vessels, and achieved 80–90% confluence. Then the isolated cells were identified for desmin and α-SMA, the markers of pericytes by immuno-fluorescence (Figure 1A).

### Characterization of Pericytes Exosomes

Exosomes derived from pericytes were analyzed by TEM, Nano-Sight particle size analysis and western blotting. Transmission electron microscopic observation showed that pericytes exosomes had the presence of spherical vesicles, with a typical cup shape. Nano-Sight particle size analysis revealed that the diameter size distribution of these nanoparticles varied from 30 to 200 nm. The specific exosomes surface markers including CD9 and CD81 were positive in pericytes exosomes according to western blotting results, which further confirmed the exosomes (Figure 1B–D).

### Pericytes Exosomes Improved Function Recovery of Mice After SCI

**FIGURE 2:**
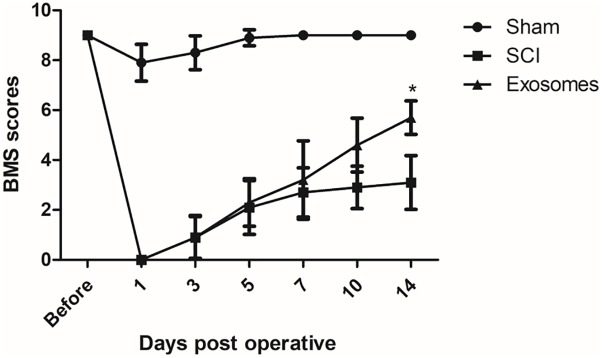
Pericytes exosomes improved functional recovery after SCI in mice. The function of hindlimb recovery was accessed from 1 day to 14 day post-operation by Basso Mouse Scale (BMS) scores. The hindlimb dysfunction was ameliorated with treatment of exosomes. Data are presented as mean + standard deviation, *n* = 5 in each group. ^∗^*p* < 0.05 compared with SCI group.

The functional recovery was observed over the next 2 weeks in all groups to determine whether exosomes treatment had rescuing effects on locomotion. As a result, exosomes treatment significantly promoted the locomotor function of hind limb from 14 days after injury when compared with that in SCI group. These results indicated that pericytes exosomes could improve the movement of mice with SCI (Figure 2).

### Pericytes Exosomes Reduces Lesion After Spinal Cord Injury

**FIGURE 3:**
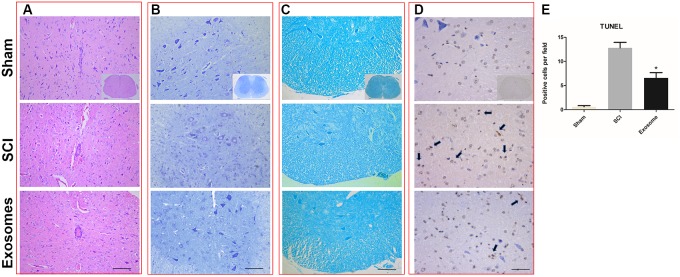
Pericytes exosomes reduced lesion after SCI. **(A)** HE staining in the three groups: Sham, SCI, exosomes. **(B)** Nissl staining in the three groups: Sham, SCI, exosomes. **(C)** LFB staining and **(D)** TUNEL staining in the three groups: Sham, SCI, exosomes. The apoptosis cells was indicated by dark color the arrow pointed. *n* = 5 in each group. Bar = 50 μm. **(E)** The number of TUNEL+ cells (cell apoptosis) in the anterior horn of the gray matter of the transverse spinal cord sections. *n* = 5 in each group. ^∗^*p* < 0.05 compared with SCI group.

The results from HE staining showed that the structure of spinal cord was complete and the morphology of neural cell was normal, the neurons were polygonal and the nuclear was large and with clear outline and there was no inflammatory cell infiltration, in the sham group. In the SCI group, spinal cord morphology was incomplete and the tissue structure was disordered. Cell nuclear split and even disappeared. The space of cells and vascular was expanded, and inflammatory cell infiltration was obvious. There was less inflammatory cell infiltration and more complete tissue structure in the exosomes group than that in the SCI group (Figure 3A).

The results from LFB showed that in the sham group, the myelin sheath was arranged neatly and with complete structure. In the SCI group, the myelin arrangement was disordered, the myelin gap was large, and the some myelin was lost. The disorder and loss of myelin sheath was improved in exosomes group compared with SCI group (Figure 3B).

The results from Nissl staining showed that in the sham group, Nissl body was neatly, well-distributed, tight and deep staining. In the SCI group, Nissl was decomposed into granules and dyed is shallow. The morphology and number of Nissl were significantly improved in exosomes group compared with SCI group (Figure 3C).

### Pericytes Exosomes Attenuated Cell Apoptosis After Spinal Cord Injury

At 48 h post-injury, TUNEL assays were applied to assess neuronal cell apoptosis in the traumatic area of the spinal cord *in vivo*. The number of TUNEL-positive cells in exosomes treated group decreased obviously when compared with that in the SCI group (Figure 3D,E). According to western blot results, treatment with exosomes attenuated Bax expression and upregulated Bcl-2 expression compared to the SCI groups (Figure 7A,B).

### Pericytes Exosomes Meliorated Microcirculation of the Spinal Cord After SCI

**FIGURE 4:**
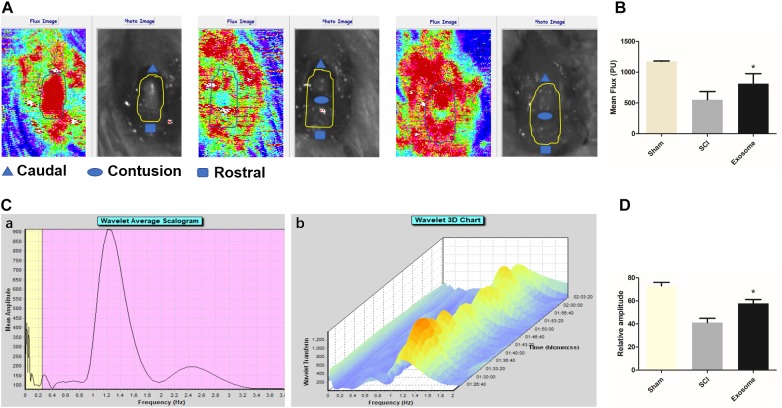
Pericytes exosomes promoted blood flow and improved endothelial function after SCI. **(A)** The blood flow of organs was detected by the Laser Doppler Line Scanner. The video image and flux image of spinal cord in the three groups: Sham, SCI, exosomes. The area of interest is circled by a yellow curve. **(B)** Quantification of blood flow in the three groups. **(C)** The vasomotion of spinal cord was measured by dual channel laser doppler monitor, and the LDF signal was analyzed by wavelet analysis. The wavelet analysis was performed by means of the Moor software. Corresponding vasomotor amplitude during study period (a). A three-dimensional (3D) plot was produced from the wavelet transformations of perfusion signals (b). **(D)** The values of the relative amplitudes of oscillatory LDF signal in the endothelial factor. *n* = 5 in each group. ^∗^*p* < 0.05 compared with SCI group.

Blood flow (BF) in the spinal cord was assessed using LDPI. A loss of microcirculation flux was observed after injury, and the reduction of perfusion signal was pronounced at the contusion site as shown in Figure 4A,B. Exosomes treatment significantly increased in perfusion at regions rostral and caudal. Additionally, the LDF investigation of microcirculation was performed by the spectral analysis of spinal cord BF oscillation (Figure 4C). The data showed that the relative value of endothelial factors decreased at the contusion site, and exosomes treatment mitigated the trend, suggesting that exosomes improved local microvascular disturbances might be related to the alteration of endothelial function (Figure 4D). The results from western blot, confirmed that the expression level of ischemia hypoxia-associated markers, HIF-1α, was obviously reduced in the exosomes group compared with that in the SCI group (Figure 7A,B).

### Pericytes Exosomes Prevented the Disruption of Blood–Spinal Cord Barrier (BSCB) and Edema Formation

**FIGURE 5:**
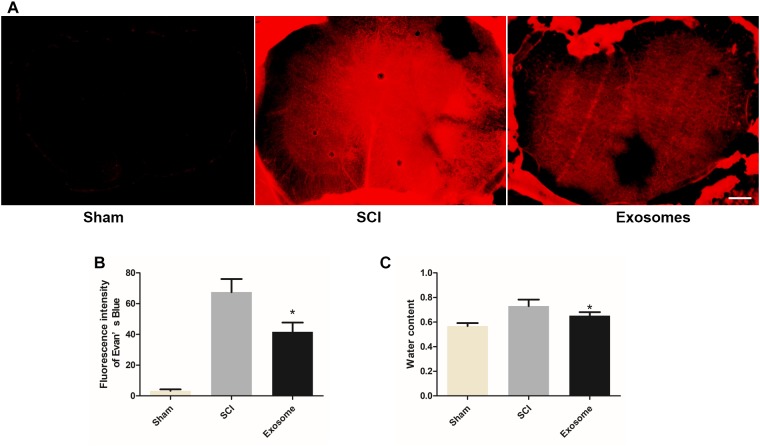
Pericytes exosomes repaired the permeability of the blood–spinal cord barrier (BSCB) and reduced edema after SCI. **(A)** Representative fluorescent image of an Evan’s Blue dye extravasation at the spinal parenchyma at 2 days after SCI. **(B)** Quantification of the fluorescence intensity of Evan’s Blue. **(C)** The water content of spinal cord in different groups. *n* = 15 in each group. ^∗^*p* < 0.05 compared with SCI group. Scale bar = 200 μm.

As shown in Figure 5A,B, after SCI, the fluorescence intensity of EB dye extravasation in damaged regions increase greatly and it reduced after exosomes treatment. In addition, exosomes effectively inhibited the increase of water content of the contusion site (Figure 5C). These results suggested that pericytes exosomes could protect against an early increase in the BSCB permeability and edema formation.

### The Effect of Exosomes on the BSCB and Edema Associated Proteins

**FIGURE 6:**
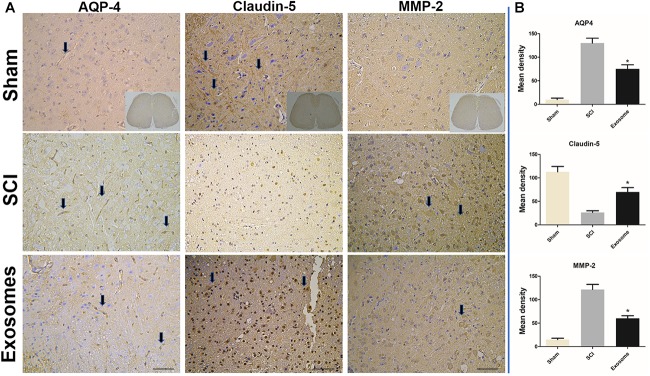
The expression of AQP-4, claudin-5, and MMP2 s was detected in sham, SCI, and Exosomes groups. **(A)** For all immunostained sections, detection of specific proteins was indicated by dark color the arrow pointed. *n* = 5 in each group. Bar = 50 μm. **(B)** Quantitative statistical analysis of protein expression of AQP-4, claudin-5, and MMP2. ^∗^*p* < 0.05 compared with SCI group.

**FIGURE 7:**
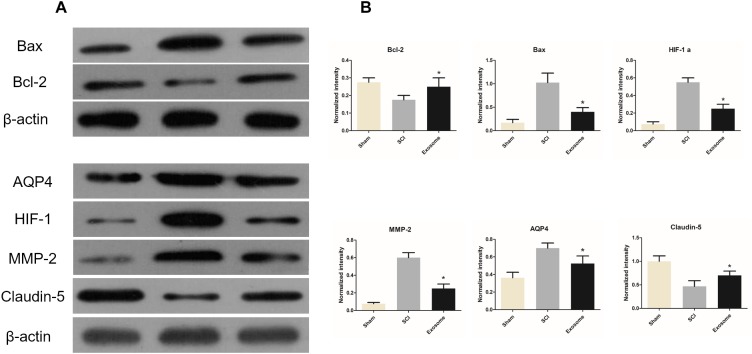
The expressions of Bax / Bcl-2 / HIF-1α / claudin-5 /MMP2 / AQP4 in three groups were determined by WB. **(A)** The expression of Bax / Bcl-2 / HIF-1α / claudin-5 / MMP2 / AQP4 in three different groups were determined by WB. **(B)** The relative expression intensity of Bax / Bcl-2/ HIF-1α / claudin-5 / MMP2 / AQP4 in three different groups. *n* = 5 in each group. ^∗^*p* < 0.05 compared with SCI group.

It was well known that the tight junction (TJ) proteins of endothelia and extracellular matrix were critical for the BSCB. Besides, aquaporins were very important in maintaining the water balance in spinal cord. The effect of exosomes on claudin-5, MMP2 and AQP4 was determined by immune-histochemistry assay. The results showed expression of claudin-5 at 48 h after SCI showed a significant disruption. However, exosomes alleviated the abnormal disruption of claudin-5. In addition, the expression of MMP2 and AQP4 in exosomes treated group decreased obviously when compared with that in the SCI group (Figure 6, 7).

### Pericytes Exosomes Protected Endothelial Cells Under Hypoxic Conditions

**FIGURE 8:**
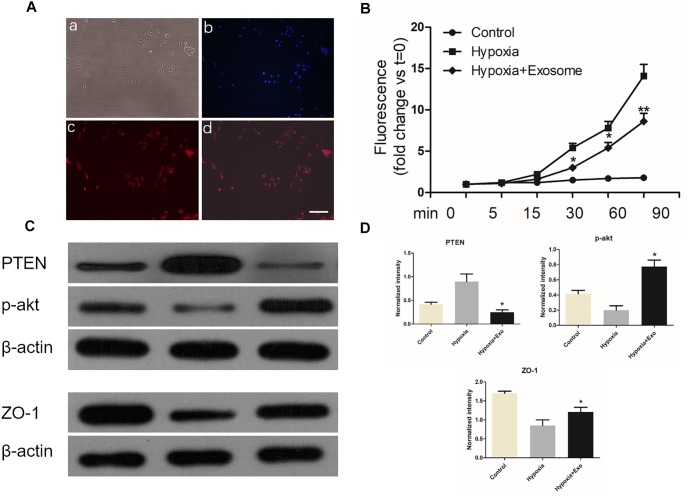
Pericytes exosomes protected endothelial cells under hypoxic conditions. **(A)** Endothelial cells were labeled with vWF, and identified by fluorescence. Bright light (a), dapi (b), vWF (c), merged (d), Bar = 100 μm. **(B)** Transwell assay was used to examine permeability of the endothelial monolayer. *n* = 5 in each group. ^∗^*p* < 0.05 compared with hypoxia group. ^∗∗^*p* < 0.01 compared with hypoxia group. **(C)** The expression of ZO-1 / PTEN / p-akt in three different groups. **(D)** The relative expression intensity of ZO-1 / PTEN / p-akt in three different groups. *n* = 3 in each group. ^∗^*p* < 0.05 compared with hypoxia group.

Endothelial cells from spinal cord microvessel were identified by labeled with vWF (Figure 8A). The permeability of endothelial monolayer was tested using *trans*-well assay. As shown in Figure 8B, the permeability of the endothelial monolayers increased greatly under hypoxic conditions. It was found that exosomes decreased the permeability induced by hypoxia. These results were in accordance with western blot results, in which TJ proteins of endothelia zonula occludens-1 (ZO-1) was alleviated by exosomes treatment. Moreover, pericytes exosomes can inhibit PTEN expression and promote p-Akt expression in endothelial cells under hypoxia (Figure 8C,D), suggesting exosomes therapy can protect the endothelial cells under hypoxia.

## Discussion

Spinal cord injury is the most serious complication of spinal trauma, which not only causes physical and mental harm to the patient, but also imposes a heavy burden on the family and society (Alilain et al., 2011). After SCI, a series of dynamic and complex pathophysiological changes occur in the injured area. It causes turbulence of microcirculation including ischemia, hemorrhage, and destruction of the blood-spinal barrier, edema and disorders of micro-hemodynamics. All these factors may affect the functional recovery by promoting apoptosis and necrosis, inflammatory cell infiltration and preventing reformation of functional synapses (Hausmann, 2003). We firstly demonstrated that exosomes from pericytes could improve blood supply, ameliorate endothelial function, protect the BSCB and reduce edema, thus leading to functional behavioral recovery in mice. Notably, our *in vitro* experiments demonstrated that exosomes can improve endothelial barrier function in hypoxic conditions, protect endothelial cells via the PTEN/Akt pathway.

The treatment of SCI was still one of the challenges in the medical field. In recent years, stem cell transplantation became more and more intensive and showed good application prospects (Wyatt and Keirstead, 2012). MSCs were a kind of pluripotent stem cells with self-renewal ability which were ideal donor cells for transplantation, because they owned neuroprotective properties and could promote functional recovery after acute SCI (Chopp et al., 2000; Varma et al., 2013). However, it showed that <1% of transplanted MSCs migrated to injured tissue. A large percentage of MSCs trapped in the lung and liver during the circulation (Phinney and Prockop, 2007).

In the past, it was thought that the repair mechanism of stem cells lies in homing and differentiation, and the replacement of damaged cells. Recently, it was considered that the extracellular vesicles secreted by transplanted cells may be more important for repairment (Ratajczak et al., 2014). Exosomes secreted by MSCs became an important active component. Studies demonstrate that exosomes of MSCs could lessen tissue damage and improve the function in various injury disease models (Théry et al., 2002; Lai et al., 2010; Camussi et al., 2013). Besides the therapeutic effects, administration of exosomes had the advantage that it could avoid limitations caused by direct stem cells transplantation (Camussi et al., 2013). Pericytes owned the characteristics of MSCs and constituted an important component of neurovascular units. They interacted with endothelial cells and took part in keeping the stability of endothelial barriers. There was no literature report on the treatment of SCI with pericytes exosomes. In the present study, we applied a series of experiments *in vivo* and *in vitro* to study exosomes treatment in the contusion SCI model. Exosomes derived from pericytes were isolated and identified with a range from 30 to 200 nm in diameter using Nano-Sight particle size analysis. They expressed the specific exosomes surface markers including CD9 and CD81. At the same time, they were confirmed by TEM.

Here, we found that traumatic injury to the spinal cord led to reduced BF and disrupted endothelial BF regulation at the contusion site. Lack of blood flow, resulting in ischemia and hypoxia, were recognized as important reasons for the failure of nervous tissue repair (Oudega, 2012). It was shown that hypoxia resulted in barrier disruptions including increased permeability, vasogenic edema, and tissue damage (Kaur and Ling, 2008), which were related with the up-regulation of HIF-1α. HIF-1α, a transcription factor, was activated by hypoxic conditions and gathered in endothelial cells. It was bound to vascular endothelial growth factor (VEGF) gene promoter and induced the expression of VEGF. The increased expression of HIF-1α were important in the adaptation of tissues and cells in hypoxic environment. In this study, our observation of blood perfusion loss and endothelium BF dysregulation after SCI suggested that the chaos of microcirculation might have contributed to delayed ischemic hypoxic tissue loss. However, the microcirculatory dysfunction was partly meliorated by pericytes exosomes treatment, which was also confirmed by our western blot results. HIF-1α levels were significantly down-regulated by pericytes exosomes. An important neuroprotective effect of pericytes exosomes may be the promotion of nervous tissue survival through the restoration of blood perfusion.

In addition, the loss of spinal cord blood perfusion following injury was mainly caused by rupture of blood vessels, which increased the permeability of BSCB with subsequent development of tissue edema. The disruption of BSCB resulted in the exchange of the harmful elements from blood and tissue and caused cell death and permanent neurological disability (Lee et al., 2012; Fan et al., 2013). Endothelial cells, pericytes, extracellular matrix, and TJs played key roles for the integrity of BSCB (Wolburg and Lippoldt, 2002). It was shown that a reduction in the permeability of BSCB attenuates edema formation after experimental contusive SCI and It was reported that reduction of edema formation after traumatic spinal injury improved functional recovery in acute and chronic phases after injury (Sharma, 2007; Fukuda et al., 2013).

Spinal cord edema can be divided into vasogenic edema and cytotoxic edema. Vasogenic edema was mainly caused by the destruction of junctions between endothelial cells, increased permeability of the BSCB, and the fluid in the blood entering the extracellular space of the parenchyma. The cytotoxic edema was mainly caused by the astrocyte metabolic disorder affecting the function of the sodium potassium pump on the membrane, which caused a large amount of water molecules to enter the cell and caused cell edema (Borgens and Liu-Snyder, 2012).

TJs around the apical end of the inter-endothelial space, were connected to adherens junctions near the basal end of the inter-endothelial space (Oudega, 2012). They were made up of zonula occludens, occludins, and claudins (Liebner et al., 2011). Claudin 5, the major claudin expressed by endothelial cell, was especially present in the BSCB (Strazielle and Ghersi-Egea, 2013). Alteration in the expression and distribution of TJs proteins was closely due to the permeability of BSCB during SCI (Liebner et al., 2011). In this study, we examined the expression of claudin-5 and found that the expression of claudin-5 was significantly enhanced in exosomes treated group when compared with that in SCI group. This implied that pericytes exosomes could promote BSCB integrity during SCI through the regulation of TJs proteins.

Matrix Metalloproteinases (MMPs) were located in the cell-surface. They were soluble and bound zinc-dependent endopeptidases that could regulate cellular infiltration, extracellular matrix degradation, release of growth factors and cytokines from the matrix, cell migration, tissue damage, remodeling, and repair (De Luca and Papa, 2017). MMPs activity was needed for the inflammatory cell infiltration and early barrier disruption after SCI. Each of these inflammatory cells expressed MMPs including MMP2. Increases of MMP2 were associated with decreases in claudin-5 in the blood vessels. MMP2 decomposed extracellular matrix (ECM), which deteriorated blood vessel damage (Noble et al., 2002). Studies demonstrated a significant up-regulation of MMPs in mouse SCI compression model (Wells et al., 2003). Moreover, it clearly showed that the pharmacological blockade of MMPs improved locomotor recovery after SCI (Hsu et al., 2006). The results also showed that pericytes exosomes treatment was involved with the decrease of MMP2 expression. It implied that the expression of MMPs in SCI might be down-regulated after treatment with pericytes exosomes, which was known to induce BSCB disruption.

Anti-Aquaporin-4 had a key role in keeping the water balance. AQP4 is widely distributed in various organs, especially in the brain and spinal cord. AQP4 was expressed on many types of cells, including glial cells, endothelial cells, and a subset of neuronal cells (Nielsen et al., 1997; Oshio et al., 2004). AQP4 has an effect on vasogenic edema and cytotoxic edema. Although AQP4 is not directly related to the formation of vasogenic edema it was essential for the removal of vasogenic edema (Fukuda et al., 2013; Hubbard et al., 2016). The high expression of AQP4 promoted the entry of liquid into cells, causing cytotoxic edema. Studies observed that a significantly better neurological recovery in AQP4 knockout mice than that in wild-type mice after SCI (Saadoun et al., 2008; Liang et al., 2015). We found that immune-reactivity of AQP4 at the injury site increased in gray and white matter at 48 h. Up-regulation of AQP4 may enhance water flow from the vasculature into spinal cord parenchyma and raise cord swelling after injury. Thus up-regulation of AQP4 represented a maladaptive response following SCI, which was also found in multiple other brain pathologies (Sun et al., 2003). We observed that pericytes exosomes could significantly alleviate the level of edema after SCI. At the same time, the expression of AQP4 also decreased.

Apoptosis was a physiological or pathological process of cells that often happened following the alteration of environmental conditions (Hengartner, 2000). Malfunction of apoptosis may be related to a large number of diseases of the CNS (Cavallucci and D’Amelio, 2011). Apoptosis affected functional recovery, nerve cell survival and axon regeneration after SCI (Zhang et al., 2015). Bax and Bcl-2 were the important molecular components about cell apoptosis. Bcl-2 was an anti-apoptotic protein and the expression of Bax represented the occurrence of apoptosis (Adams and Cory, 1998). After neuronal injury, the pro-apoptotic proteins Bax was shown to be upregulated, while the anti-apoptotic protein Bcl-2 was down-regulated (Seki et al., 2003). TUNEL assay demonstrated that treatment with pericytes exosomes could alleviate the apoptosis of neuronal cells in the SCI model, which was also confirmed by the results from western blot. The level of Bax was significantly repressed by pericytes exosomes, whereas the level of Bcl-2 was up-regulated. Both results indicated that administration of pericytes exosomes could protect neuronal cells from injury-induced apoptosis.

To further explore this barrier protection mechanism, we evaluated the extent of permeability of endothelial cells by Transwell system in the hypoxia model *in vitro*. As expected, the *trans*-well assay confirmed that pericytes exosomes could effectively prevent endothelial barrier and up-regulate the expression level of junction protein ZO-1 in the hypoxia condition, which further confirmed our results *in vivo*.

Phosphatase and tensin homolog deleted on chromosome 10 (PTEN) was a tumor suppressor gene. PTEN was considered as a negative regulator of PI3K/AKT pathway. PI3K/AKT pathway has been extensively studied as a regulator of cell survival and apoptosis. p-Akt is phosphorylated protein kinase B (p-PKB), an important signal protein molecule, which can inhibit cell apoptosis and promote cell survival (Bsibsi et al., 2006; Farina et al., 2007). In this article, western blot results showed pericytes exosomes could inhibit PTEN expression and promote p-Akt expression in SMECs, which suggested exosomes therapy could promote the survival of endothelial cells under hypoxia and reduce cell apoptosis.

## Conclusion

**FIGURE 9:**
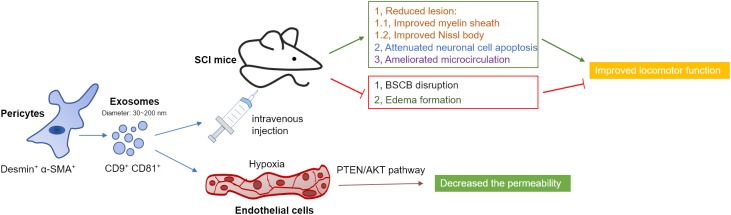
Diagram illustrated the proposed mechanism of treatment of pericytes exosomes after traumatic SCI in mice. Pericytes exosomes improved myelin sheath and Nissl body, attenuated cell apoptosis, ameliorated microcirculation, suppressed BSCB disruption and edema formation and eventually improved functional behavioral recovery after SCI. Especially, treatment of pericytes exosomes protected the barrier of spinal cord microvascular endothelial cells under hypoxia condition, which was related to PTEN/AKT pathway.

In conclusion, the present study we demonstrated that treatment with pericytes exosomes could promote blood flow, improve endothelial function, protect the BSCB, alleviate the apoptotic response, and thus promote functional and behavioral recovery after SCI (Figure 9). In particular, one of the underlying mechanisms may be the protection of the endothelial cells in hypoxia condition. These findings suggested that pericytes-derived exosomes is a potential new therapeutic interventions for SCI.

## Ethics Statement

The procedures of animal experiments were approved by the Experimental Animal Care and Ethics Committee of the Institute of Microcirculation, Chinese Academy of Medical Sciences (CAMS) and Peking Union Medical College (PUMC).

## Author Contributions

XY, QW, and PW substantial contributions to the conception or design of the work, acquired, analyzed, or interpreted the data for the work. YJ, HY, and YT drafted the work or revised it critically for the important intellectual content. All authors approved the final version to be published. ZL, HZ, and RX agreed to be accountable for all the aspects of the work in ensuring that questions related to the accuracy or integrity of any part of the work are appropriately investigated and resolved.

## Conflict of Interest Statement

The authors declare that the research was conducted in the absence of any commercial or financial relationships that could be construed as a potential conflict of interest.
